# Isolating, characterising and identifying a Cry1Ac resistance mutation in field populations of *Helicoverpa punctigera*

**DOI:** 10.1038/s41598-018-21012-w

**Published:** 2018-02-08

**Authors:** Tom Walsh, Bill James, Maissa Chakroun, Juan Ferré, Sharon Downes

**Affiliations:** 1 CSIRO, Black Mountain Laboratories, Canberra, ACT 2601 Australia; 2 ERI of Biotechnology and Biomedicine (BIOTECMED), Universitat de València, Burjassot, 46100 Spain; 3 CSIRO, Myall Vale Laboratories, Kamilaroi Highway, Narrabri, NSW 2390 Australia

## Abstract

Transgenic cotton expressing insecticidal proteins from *Bacillus thuringiensis* (Bt) has been grown in Australia for over 20 years and resistance remains the biggest threat. The native moth, *Helicoverpa punctigera* is a significant pest of cotton. A genotype causing resistance to Cry1Ac in *H. punctigera* was isolated from the field and a homozygous line established. The phenotype is recessive and homozygous individuals possess 113 fold resistance to Cry1Ac. Individuals that carry Cry1Ac resistance genes are rare in Australia with a frequency of 0.033 being detected in field populations. RNAseq, RT-PCR and DNA sequencing reveals a single nucleotide polymorphism at a splice site in the *cadherin* gene as the causal mutation, resulting in the partial transcription of the intron and a premature stop codon. Analysis of Cry1Ac binding to *H. punctigera* brush border membrane vesicles showed that it is unaffected by the disrupted *cadherin* gene. This suggests that the major Cry1Ac target is not cadherin but that this molecule plays a key role in resistance and therefore the mode of action. This work adds to our knowledge of resistance mechanisms in *H. punctigera* and the growing literature around the role of cadherin in the mode of action of Cry1 type Bt proteins.

## Introduction

Transgenic cotton, *Gossypium hirsutum* L. varieties expressing *Bacillus thuringiensis* (Bt) toxins have been grown around the world since 1996^1^. In Australia, the first generation of transgenic cotton contained the insecticidal toxin Cry1Ac and subsequent dual gene varieties, introduced in 2004 contained both Cry1Ac and Cry2Ab. A three gene variety introduced in 2016 contains Cry1Ac, Cry2Ab and Vip3A^2^. The stacked toxin varieties are expected to increase the longevity of transgenic technology, delaying the development of resistance in pest species of Lepidoptera. The major international cotton pest *Helicoverpa armigera* is a recidivist at evolving resistance to synthetic pesticide sprays and a similar propensity to render Bt toxins harmless is expected^3,4^. In contrast *Helicoverpa punctigera*, endemic to Australia, has failed to develop field relevant resistance to any synthetic pesticide sprays and therefore is considered a reduced threat to transgenic cotton^5,6^. Both *H. armigera* and *H. punctigera* are effectively controlled by Bt toxins and no field failures in Bt cotton have been reported in Australia^2,7^.

The mode of action of Bt toxins involves proteolytic processing in the insect midgut and binding to several proteins in the brush border membrane, which leads to the formation of pores causing paralysis of the midgut and the eventual death of the insect larva. Alkaline phosphatase (ALP), several aminopeptidase N proteins (APN), cadherin (CAD) and the ABCC transporters have all been implicated as surrogate receptors for Cry1 proteins in various insect species (reviewed in Wu^8^). Disruption of any of these interactions with the Cry1 protein can substantially reduce the sensitivity to the Bt protein, as has been shown in several species where binding of the Bt toxin has been shown to be drastically reduced in the resistant insects (reviewed in Ferre and Van Rie^9^)^10^.

The ecology of *H. punctigera* is similar to that of *H. armigera*, with both species having a broad host range with the potential to cause significant damage to a number of crops^11^. *H. punctigera* is thought to migrate from inland Australia to the cropping regions in Eastern Australia. This lack of an overwintering population and a regular influx from non-agricultural areas was thought to contribute to the lack of phenotypic resistance to pesticide sprays observed in this species despite experiencing similar selection pressures to *H. armigera*. Recently some doubt was cast on this relationship which is complicated by a lack of information on its patterns of movement and overwintering capacity within the Australian cropping system^12^.

While it was thought that *H. armigera* posed a greater threat than *H. punctigera* to the continued effectiveness of Bt proteins, in both species resistance alleles have been detected to all three Bt proteins currently deployed. Indeed background resistance allele frequencies to Cry2Ab and Vip3A are at least 0.01 and pre-date the commercialisation of cotton expressing these Bt proteins^13–15^. From 2004 until 2011 there was some evidence of increases in resistance allele frequency in *H. punctigera* to Cry2Ab and since then the allele frequencies have not increased further^2,16^. The Cry1Ac toxin has been deployed in commercial varieties of cotton since 1996 (22 years), and while there is no evidence of cross-resistance between Cry1Ac and Cry2Ab or Vip3A in Australian target pests, there may be opportunities to select for resistance at least to the former due to exposure at a sub-optimal dose later in the growing season. For instance, larvae of *H. armigera* and *H. punctigera* typically survived on post-squaring plants of the first generation single toxin Cry1Ac cotton due to poor expression, and for short periods post-squaring can develop to adults on dual-toxin Cry1Ac and Cry2Ab cotton presumably due to sub-optimal expression^17^. Both of these scenarios represent opportunities for resistance to Cry1Ac to be selected. There may be similar opportunities for selection against Vip3A^18^.

As part of a resistance management plan (RMP) adopted by Australian growers for Bt cotton, a project was established in 2003 to monitor resistance in *H. armigera* and *H. punctigera* using F_2_ screens to detect rare alleles and potentially isolate resistant colonies for further research^19^. Until 2012 only three and five instances of Cry1Ac resistance alleles were detected respectively in *H. armigera* (n = ~5500) and *H. punctigera* (n = ~6500) suggesting that they are extremely rare (see also Downes and Mahon 2012^20^). In both species, the original field collected insects were heterozygotes. From one of these detections isolated in the 2009/10 season a resistant colony of *H. punctigera* was established (hereafter Hp9-3784) which enabled research into the current frequencies of this type of resistance and its characteristics. Our characterisation work included studies of Cry1Ac binding affinity to brush border membrane vesicles in the Cry1Ac resistant and susceptible strains of *H. punctigera*, and a molecular-based approach which allowed us to identify the gene as well as the genetic mutation responsible for the observed resistance.

## Methods

### Insect rearing and bioassay protocol

The rearing methods used to maintain *H. punctigera* were the procedures described by Teakle and Jensen (1985) except propionic acid (0.08%) was substituted for formalin. Rearing trays (Oliver Products Company, Grand Rapids, MI, USA) were covered and heat-sealed by a perforated lid. Moths were provided with a 4% honey/sugar solution, and housed in containers that were open at the top and covered with nappy liners secured around their lip. To reduce the risk of cross-contamination work benches and apparatus were cleaned between handling different colonies. Susceptible and resistant colonies were reared in different rooms.

The Cry1Ac protein used in F_2_ crosses to isolate resistant alleles from the field was produced from a Bt strain HD73 as described in Akhurst *et al*.^21^. The same method was used to generate toxin for use in bioassays and to determine the inheritance of resistance.

Insects were exposed as neonates to Cry1Ac on the surface of the diet. Stock suspensions of the proteins were made and diluted with water to generate the appropriate concentrations and distilled water was used as a control. The bioassays were conducted in 45 well plates, one neonate per well and covered with breathable film and mortality (no response to manual stimulation) was assessed after 7 days under the same conditions used for rearing.

### Insect strains and phenotypes

The general laboratory strain (designated HPM) used in our assays is susceptible to Cry1Ac toxins which was monitored regularly by evaluating responses to discriminating doses of this toxin that kills ~95% of susceptible neonate larvae. It was created in 2010 by bulk mating 5–6 individuals from 40 iso-female families that scored negative for Cry1Ac, Cry2Ab and Vip3A resistance in F_2_ screens^22^.

The resistant strain, Hp9-3784, was established from a single *H. punctigera* pair collected as eggs on Bollgard II cotton in the Gwydir Valley, NSW, in the 2009/10 season. Progeny from the pair were allowed to mate amongst themselves and the Hp9-3784 colony was formed from F_2_ offspring that survived a discriminating concentration of Cry1Ac toxin (μg/cm^2^) applied as a surface treatment. These F_2_ screens were performed with the specific intention to detect resistance to Cry toxins in *H. punctigera* (see Downes *et al*.^13^ for further details). Preliminary bioassays showed that the Hp9-3784 colony established from survivors of the discriminating dose used in the F_2_ screen also survived the discriminating concentration of Cry1Ac, and we assumed that it was homozygous for the gene conferring resistance to Cry1Ac. The susceptible strain, HPM was assumed to be homozygous susceptible. The F_1_ produced by crossing Hp9-3784 and HPM were assumed to be heterozygotes.

Since Hp9-3784 initially possessed a very restricted gene pool it was crossed to HPM 3 times at generations 5, 10 and 13 (with field collections deemed F_0_). Following each cross, the colony was maintained without selection for one generation and re-selected with Cry1Ac toxin (as above) as a diet surface contamination treatment. Subsequent generations were selected at this dose. This method maintained fitness in Hp9-3784. Since this colony was backcrossed to the HPM strain on three occasions, the two strains would be expected to share 87.5% of their genetic background. Most of the assays reported here were performed with individuals from the near-isogenic 3^rd^ outcross (generations 15 to 21) to reduce the potentially misleading effects of hybrid vigour that may be evident when crossing colonies of *H. punctigera*.

### F_1_ screens for Hp9-3784-like resistance

To estimate frequencies of Hp9-3784-like alleles conferring resistance to Cry1Ac in *H. punctigera* from 2013/14 until 2015/16 we used F_1_ screen methods reported previously^23^. This work was conducted in our Narrabri laboratories as part of the Bt resistance monitoring program supported by the Australian Cotton Industry^19^.

*H. punctigera* were sampled from a range of cultivated and uncultivated hosts in the major cotton regions in eastern Australia. Field-collected eggs were reared to pupae and males and females were placed in groups in separate cages. As moths emerged, individual males were placed with a virgin female from the Hp9-3784 colony. Similarly, individual females were placed with a male from the Hp9-3784 colony. If fertile eggs were obtained from such crosses, F_1_ offspring were exposed to toxin at a dose that would permit only homozygous resistant insects to grow to at least 3^rd^ instar by 7 days. If the field-derived individuals were heterozygous for an allelic resistance mechanism, we would expect that approximately 50% of the larvae to be homozygous for resistance and therefore to thrive. In the unlikely event that we collected and tested homozygotes from the field, the frequency of survivors would be close to 100%.

We used Bayesian inference to estimate the expected allelic frequency and the 95% credibility intervals for F_1_ screens. The expected Bt resistance allele frequency in the population was estimated using equation 4 from Yue *et al*.^24^. Bayesian methods determine the probability that the present experimental results give an estimated *p* within some credibility of the parameter estimate. The 95% credibility intervals for our estimated frequencies were determined using equation 15 from Andow and Alstad^25^.

### Genetic characterisation of resistance

The HPM and Hp9-3784 strains were sexed at the pupal stage and reciprocal crosses were produced. The F_1_ hybrids were reared through to adults and backcrossed as above. Neonates were tested by surface application as described above. Each bioassay was repeated 3–5 times and the LC50 was calculated from probit analysis using POLO-PC software (LeOra Software). The resistance ratio was expressed as the ratio of the LC50 of Hp9-3784 to HPM.

### Identification of candidate genes

Total RNA was extracted from midguts of 5 larval stage four *H. punctigera* and mRNA sequencing was performed. The *H. punctigera* midgut transcriptome was sequenced using Illumina PE sequencing and reads were assembled using Trinity^26^ at BGI. Briefly, assembled transcripts were annotated using BlastX (evalue < 0.00001) against protein databases (Nr, SwissProt, KEGG, COG). Gene ontology (GO) annotation was obtained for each unigene using Blast2GO. Candidate resistance genes were identified by reciprocal Blastx using the *H. armigera* homologues^27^.

Primers (Supplementary Table 1) were designed on the candidate gene and RT-PCR was performed on oligo dT primed reverse transcribed cDNA (SuperScript III, Life Technologies, CA USA; Standard Taq, NEB, Ipswich, MA, USA). Primers for the gDNA were designed on the *H. punctigera* cDNA and the *H. armigera* genome^27^ to avoid introns. PCR products were sequenced by Sanger sequencing and assembled to the reference sequences using CLCgenomics (v 9.0) workbench (Qiagen, Netherlands).

In order to confirm the presence of the mutation, crosses of Hp9-3784 with susceptible HPM were generated and the F1 generation was selfed to produce the F2 generation containing a known frequency of homozygous resistant and heterozygous individuals. The frequency of the putative resistance allele was tested using specific primers on the genomic DNA (Supplementary Table 1).

### Cry1Ac binding to *H. punctigera* brush border membrane vesicles (BBMV)

Midgut isolation, BBMV preparation, Cry1Ac radiolabelling and binding assays were performed as described previously^28^. The estimated specific activity of the labelled protein was 6.0 mCi/mg. After terminating the reaction, the radioactivity retained in the pellet was measured in a model 2480 WIZARD^2^ gamma counter. Binding reactions were performed with 0.02 nM ^125^I-Cry1Ac in 0.1 ml final volume of binding buffer (8 mM Na_2_HPO_4_, 2 mM KH_2_PO_4_, 150 mM NaCl; pH 7.4; 0.1% bovine serum albumin) for 60 min at 25 °C. Non-specific binding was determined by adding 2000-fold of unlabelled Cry1Ac. Competition experiments were performed by incubating 20 µg/ml of BBMV, from both the susceptible and resistant colonies, in the presence of an increasing amount of unlabelled Cry1Ac. The dissociation constant (*K*_*d*_) and the concentration of binding sites (*R*_*t*_) were calculated using the LIGAND program^29^.

### Data availability

The raw data used to support the findings of this study and the supplementary information are available from the corresponding author upon reasonable request.

## Results

### Frequencies and characterisation of Hp9-3784-like Cry1Ac resistance

The F_1_ tests performed in 2013/14, 2014/15 and 2015/16 estimated Hp9-3784-like Cry1Ac resistance alleles in *H. punctigera* to be 2 of 498, 1 of 394 and 6 of 828 respectively. The frequency of detection of positive alleles do not differ significantly between seasons (X^2^ = 1.34, df 2, P = 0.51). Pooling the data from the three seasons yields a frequency of Hp9-3784-like Cry1Ac resistance alleles in *H. punctigera* of 0.033 with 95% credibility intervals of 0.021 and 0.047. In 2015/16 two of the reported alleles were present in the same homozygous resistant individual.

**Figure 1:**
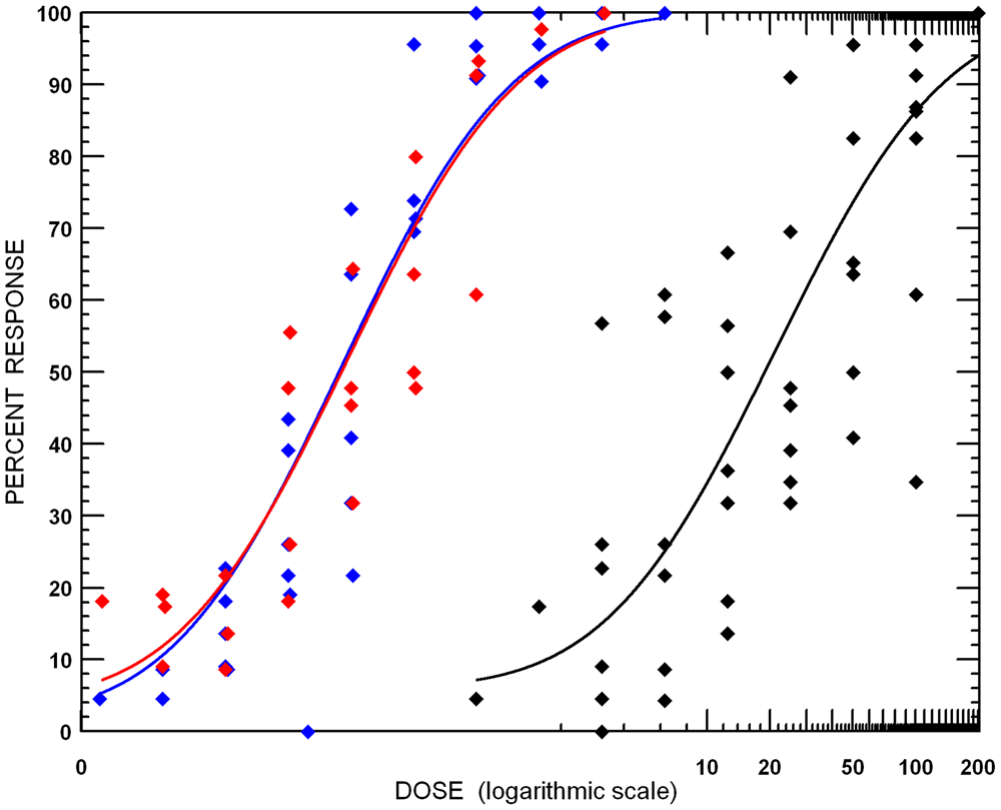
Bioassay results of Cry1Ac resistant, susceptible and heterozygotes exposed to doses of Cry1Ac. The Cry1Ac resistant Hp93784 is 113 fold more resistant to Cry1Ac than both the susceptible and heterozygote individuals. Hp9-3784 - black line and points, HPM – blue line and points, F_1_ heterozygotes – red line and points.

The bioassays of HPM, Hp9-3784 and the F_1_ hybrid suggests that the resistance is recessive and mediated by one major gene (Fig. 1). The LC_50_ values for HPM, the F_1_ hybrid and 9-3784 were 0.21, 0.31, 23.75 ug cm^−2^ respectively giving a resistance ratio of Hp9-3784 to HPM of 113 fold. The susceptibility of the F_1_ heterozygote was the same as that of HPM suggesting that this resistance is completely recessive. Backcrossing the F_1_ hybrid back to Hp9-3784 also confirmed the recessive nature of the resistance, displaying an intermediate susceptibility (LC_50_ = 2.08 ug cm^−2^). This result also suggests a single gene is responsible for resistance to Cry1Ac in *H. punctigera*.

### Identification of candidate genes in *H. punctigera*

To identify and design primers on known candidates for Cry1Ac resistance, 4GB of transcriptome sequence was generated from midguts of 5 pooled 4^th^ stage *H. punctigera* HPM larvae. Summary data on the assembly and the annotated transcriptome can be found in Supplementary Figure 1. Assembled transcripts were screened and then identified as potential targets for Cry1Ac using genes known to cause resistance in other organisms. Candidate genes (*cadherin*, *APN1, APN2 and APN3* and *ABCC2*) were all identified by Blastn using homologous sequences from *H. armigera* or existing sequences in the database (Table 1). A mutation was identified in cadherin where a disrupted transcript was observed in Hp9-3784 (Supplementary Data 1). A phylogenetic tree of the cadherin genes from a number of related organisms was constructed and places the cadherin sequence from *H. punctigera* (MF929076) as most closely related to that of *H. armigera* (Supplementary Figure 2).

**Table 1:** Cry1Ac resistance related genes examined in *Helicoverpa punctigera*.

Gene Name	NCBI number	*H. armigera* homologue
Cadherin	MF929076	XP_021195552.1
CadherinR	MF929078	—
HpABCC2	Applied for	XP_021200557.1
HpAPN1	AF217248.1	XP_021192756.1
HpAPN2	AF217249.1	XP_021192757.1
HpAPN3	AF217250.1	AY279537.1

**Figure 2:**
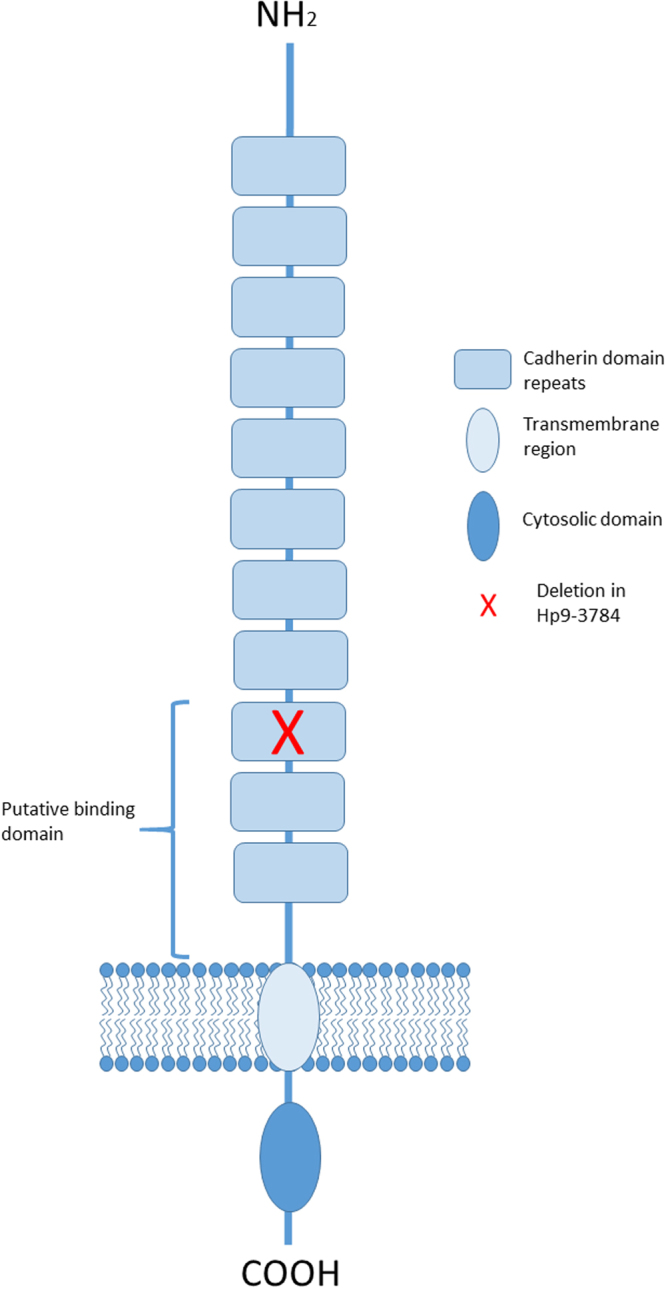
A schematic representation of the cadherin protein in *H. punctigera* and the location of the mutation identified in Hp9-3784. The binding domain would seem to be disrupted but binding does not change suggesting that Cry1Ac is binding to a different receptor in the insect midgut.

A 58 bp insertion in the cDNA sequence of the *cadherin* gene was identified in the sequenced cDNA from Hp9-3784 as compared to HPM (Fig. 2). This insertion disrupts the coding sequence in cadherin domain 9 causing a downstream frame shift and a premature stop codon for the rest of the protein. This would result in a truncated protein of 1243 amino acids without the putative binding domain, the membrane anchoring domain, presumably retained inside the cell and not exposed to the Cry1Ac, or alternatively, exported into the gut where it would be degraded (Supplementary Figure 3). The 58 bp insertion most closely matched intron sequence from the *H. armigera* cadherin gene (AY714876.1). Sequencing of the *H. punctigera* gDNA confirmed that the insertion in the cDNA is intron sequence. The read through appears to be the result of a single nucleotide polymorphism (T/A) in the genomic DNA which removes the splice site (Supplementary Figure 4).

Diagnostic PCR primers were designed for both cDNA and gDNA (Supplementary Table 1). The cDNA test requires a simple PCR amplification of the transcript around the region of the insertion in the cDNA and identification of the resistance locus by gel electrophoresis. Identifying this mutation in the gDNA requires DNA sequencing to identify the specific single base pair mutation at the intron-exon boundary. This genomic DNA test was applied to the genetic crosses to show the association of the identified mutation with the resistant phenotype and the association was 100% (unselected n = 18, selected n = 24, X^2^ = 12, df 2, P = <0.05).

### Binding of ^125^I-Cry1Ac to BBMV from last instar larvae

To determine whether the mutation in the cadherin gene gave rise to a reduction of Cry1Ac binding to the resistant insects midgut epithelium, this protein was labelled with ^125^I and its binding to BBMV from the susceptible (HPM) and the resistant (Hp9-3784) colonies was tested. In a first approach, a fixed concentration of labelled protein was incubated with increasing concentrations of BBMV from each strain (Fig. 2). In the two colonies, an increase in the specific binding of ^125^I-Cry1Ac was observed, corresponding to the increase of BBMV concentration. The specific binding was not substantially different for the susceptible and resistant insects.

**Figure 3:**
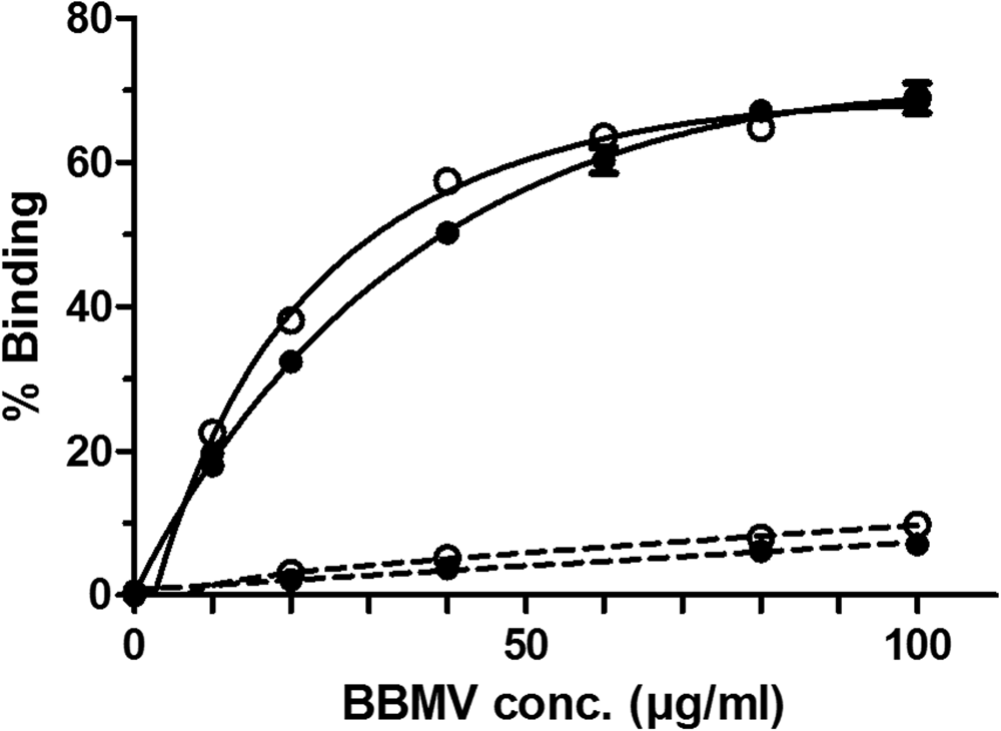
Assay for specific binding. Binding of ^125^I-Cry1Ac to BBMV from the susceptible HPM (full circles) and resistant Hp9-3784 (open circles) *H. punctigera* colonies at increasing concentrations of BBMV. Total binding (solid line) and non-specific binding (broken line) from duplicate data points is represented. The specific binding is the difference between total and non-specific binding. Each data point represents the mean of two replicates.

Since resistant insects bound Cry1Ac specifically, to see whether this binding differed quantitatively from that of susceptible insects, competition assays were carried out using a fixed amount of ^125^I-Cry1Ac and BBMV, and increasing concentrations of unlabelled Cry1Ac. The competition curves showed no major differences between BBMV from the two colonies (Fig. 3). The dissociation constant (*K*_*d*_) and the concentration of binding sites (*R*_*t*_) values, obtained from the competition curves, were very similar, with almost the same *R*_*t*_/*K*_*d*_ ratio, which indicates a similar overall binding affinity (Table 2).

**Table 2:** Equilibrium dissociation constant (*K*_*d*_) and concentration of binding sites (*R*_*t*_) of Cry1Ac with *H. punctigera* BBMV from the susceptible (HPM) and resistant (Hp9-3784) colonies.

Colony	Mean ± SEM		
	*K*_*d*_ (nM)	*R*_*t*_ (pmol/mg)	*R*_*t*_/*K*_*d*_
HPM	1.85 ± 0.15	43.7 ± 2.2	23.6
Hp9-3784	1.00 ± 0.19	22.1 ± 2.4	22.1

## Discussion

Genetically modified crops expressing Bt proteins have successfully controlled lepidopteran pests^7^. However, as with all other pesticides, the evolution of resistance remains a risk to their continued effective use. Cry1Ac was the first, and remains the most widely used, Bt protein expressed in transgenic plants to control Lepidoptera. Resistance to this toxin is affecting the efficiency of transgenic cotton against *H. armigera* in China^30^, *H. zea* in USA^31^ and *P. gossypiella* in India^32^. Monitoring populations for resistance is a vital component of a resistance management plan that aims to enable farmers to respond to signs of emerging threats. In Australia, F_2_ and F_1_ screens against Cry1Ac toxin with *H. punctigera* were used between 2003–2012 and 2013–2015 respectively^2^. The additional data contributed in this study from the F_1_ screens suggests that resistance to Cry1Ac in *H. punctigera* remains rare.

While it is not possible in our study to directly compare frequencies obtained during the same season with F_2_ vs F_1_ screens, it is worth noting that the resistance allele frequency from the F_2_ tests for Cry1Ac resistance reported previously was significantly lower than the F_1_ test results described in this manuscript using the Hp9-3784 strain. The F_2_ tests identified 5 alleles in over 6000 tested over nine years whereas the F_1_ test identified 9 alleles from 1720 tested in three years. The same phenomenon has been observed for Cry2Ab resistance in *H. armigera*^33^. In that case the authors speculated that the simplicity of the F_1_ test made it inherently less susceptible to mortality effects or the impact of laboratory acclimatisation and they concluded that it was likely the more reliable test of field allele frequencies. Another possible explanation lies in the mechanism of resistance identified in this work with fitness costs often observed in Cry1 resistances which would likely be more relevant to the multi-generation F_2_ screen^34,35^. Another possibility is that the higher frequencies reported from the more recently performed F_1_ screens reflect a real increase over time however this is at odds with the lack of significant difference among seasons detected for the three years of F_1_ screening. Further work to identify the specific alleles that have been isolated using the F_1_ test will shed light on the diversity of resistance alleles and potentially provide evidence for selection of a specific allele from background variation.

The mechanism of Cry1Ac toxicity is thought to be a multi stage process involving a number of different genes (reviewed by Wu^8^). In almost all models for the mode of action of Cry1 toxins, cadherin plays a role, initially as a reversible binding site which is thought to enhance the formation of the toxin oligomers which are the most effective structures for forming pores. It appears that Bt toxins will form pores in the absence of cadherin but much more slowly and this potentially explains the dose dependant effect of the resistance observed in this work and in others where resistance can be overcome by the addition of more toxin.

Of the several candidate genes coding for proteins that have been described as receptors for Cry1Ac^36^, we found that a disruption of the cadherin gene is the likely resistance mechanism for Cry1Ac in *H. punctigera* in Australia. Disruption of the cadherin gene can occur in a number of different ways and the mutation described in cadherin in *H. punctigera* is consistent with those found in Cry1Ac resistant *H. armigera* from China^37,38^. In these examples, a mutation disrupting the formation of the mature protein occurs, disrupting the role cadherin plays in the toxicity of Cry1Ac. There is no large insertion or deletion in the genome but a simple single nucleotide polymorphism that disrupts splicing and results in a transcribed intron. Disrupted and alternative splicing has also been associated with Cry1Ac resistance in in other species^39^. The mutation in the Hp9-3784 strain produces a truncated protein that lacks the transmembrane region and is likely retained inside the cell.

**Figure 4:**
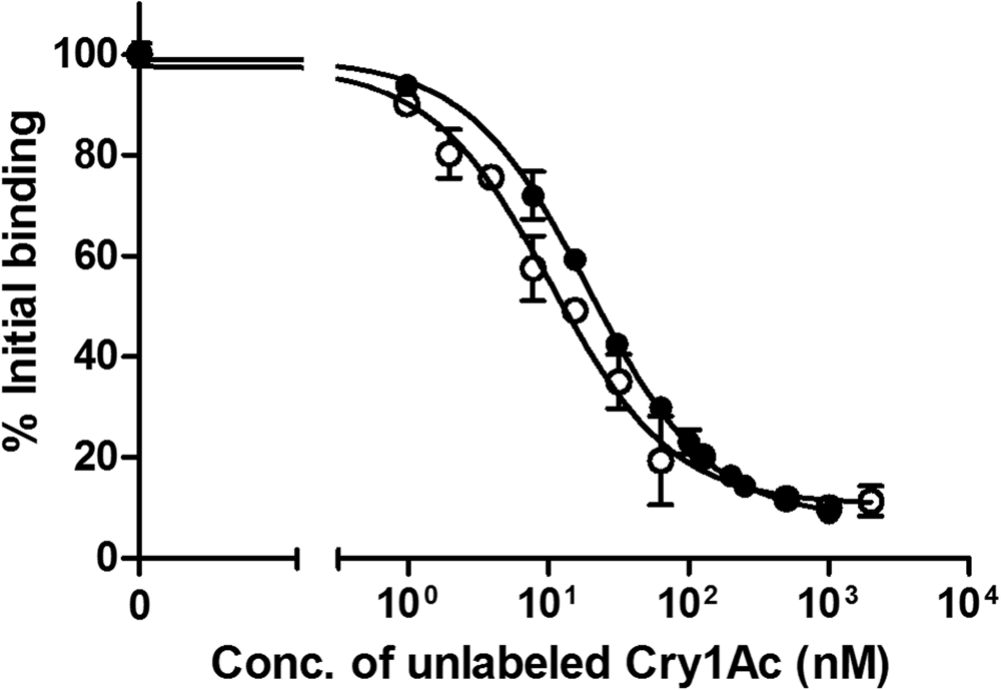
Competition binding assay. Binding of ^125^I-Cry1Ac, at increasing concentrations of unlabelled Cry1Ac, to BBMV of the susceptible HPM (full circles) and resistant Hp9-3784 (open circles) *H. punctigera* colonies. Each data point represents the mean of two replicates.

Since cadherin is a receptor for Cry proteins in many insect species^40^, we wanted to check whether the mutation in the Hp9-3784 strain would have an effect on Cry1Ac binding. The results (Figs 3 and 4) showed that BBMV from resistant insects bound Cry1Ac specifically and that no major differences in binding parameters were found between resistant and susceptible insects. This result is in agreement with those previously reported in *Heliothis virescens* showing that mutations resulting in lack of cadherin in the midgut membrane do not affect Cry1Ac binding^41,42^. In *H. armigera*, similarly to our results, Xu and Wu^43^ found that Cry1Ac bound with similar affinity to BBMV (and the concentration of binding sites was not significantly different) from Cry1Ac-susceptible and -resistant insects, the latter lacking the cadherin receptor. It is long known that Cry1Ac has three binding sites in *H. virescens* and that only site A is responsible for resistance to this toxin^44^. Therefore, our results suggest that the mode of action of Cry1Ac in *H. punctigera* follows a similar pattern to that in other heliothine species and that the role of cadherin is probably to speed up oligomer formation but it is not the target site for Cry1Ac binding. Thus, a mutation in the *cadherin* gene would confer resistance by slowing down the effect of Cry1Ac without reducing its binding to the midgut epithelium.

Our results support the growing evidence that mutations causing lack of cadherin in the midgut epithelial membrane do not affect binding of Cry1Ac and that the lack of Cry1Ac binding observed in some resistant strains is mainly due to mutations in the ABCC transporters. Gahan *et al*.^42^ tested Cry1Aa, Cry1Ab, and Cry1Ac binding to BBMV from different Cry1Ac resistant strains and showed that lack of cadherin (the mutation in strain YFO) did not affect Cry1Ab or Cry1Ac binding, whereas binding of Cry1Aa was strongly reduced. Conversely, Cry1Ab or Cry1Ac did not bind to BBMV from insects (strains YEE and YHD3) carrying a mutation resulting in lack of the ABCC2 transporter, though Cry1Aa did. In non-heliothine species, *Plutella xylostella* and *Trichoplusia ni*, reduced binding of Cry1Ac^45,46^ was correlated with mutations in the ABCC2 transporter^47^, but not in the cadherin gene^48,49^. In *Pectinophora gossypiella*, mutations in the *cadherin* gene^50^ did not cause reduction in Cry1Ac binding^51^. Based on results from experiments with Sf9 cell lines expressing the *H. virescens* cadherin, the ABCC2 transporter or both, the authors propose that the ABCC2 transporter is the target for Cry1A toxins and that cadherin plays a “supporting role in increasing Cry1A toxicity” by helping oligomerization of the monomeric toxins^52^. Lack of binding of Cry2Ab to resistant *H. armigera* with a mutation in the ABCA2 transporter has also been reported^28,53^. All of this information suggests that Cry1Ac binding to cadherin is transient and speeds up oligomerization of the toxin, which makes the oligomer readily bind to the final receptor promoting pore formation.

The molecular characterisation of the mechanism of Cry1Ac resistance in *H. punctigera* has implications for how the risk of resistance developing to Cry1Ac is managed. These results show that, as found in other species, resistance to Cry1Ac in *H. punctigera* is caused by a mutation in the *cadherin* gene. With this knowledge we can extrapolate from examples in other species where this type of mutation has been identified. In other regions and species, there are increasing reports of Cry1A type resistance, particularly in *H. armigera* and often mediated by mutations in the *cadherin* gene. Of particular concern is the discovery of a non-recessive mutation in China^38^ as while most of the known cadherin mutations present as recessive, a dominant genotype would more rapidly spread through the population. Cadherin mutations in *H. punctigera* are present in the population but there is no evidence of an increase in frequency or any observed dominance despite 20+ years of selection pressure.

When cotton expressing Cry1Ac was originally introduced there was a restriction that no more than 30% of the cotton crop could be grown to this variety. This, combined with the characteristics of this resistance, recessive and rare and conceivably with a fitness cost (S. Downes, unpublished data), may have kept selection pressure low in the initial stages before the introduction of dual gene cotton (Cry1Ac and Cry2Ab) to contribute to the continued success of transgenic cotton and resistance management in Australia.

## Electronic supplementary material

Supplementary data
