# Supplementary material for: Isolating, characterising and identifying a Cry1Ac resistance mutation in field populations of Helicoverpa punctigera
Source: Sci Rep. 2018 Feb 8;8:2626. doi: 10.1038/s41598-018-21012-w (PMC5805676; doi:10.1038/s41598-018-21012-w)
Supplement: Supplementary file 1 — Supplementary data [file 41598_2018_21012_MOESM1_ESM.doc]

Isolating, characterising and identifying a Cry1Ac resistance mutation in field populations of *Helicoverpa punctigera*.

Tom Walsh^1^*, Bill James^1^, Maissa Chakroun^2^, Juan Ferré^2^, Sharon Downes^3^

^1^CSIRO, Black Mountain Laboratories, Canberra, ACT, 2601, Australia

^2^ERI of Biotechnology and Biomedicine (BIOTECMED), Universitat de València, 46100 Burjassot, Spain

^3^CSIRO, Myall Vale Laboratories, Kamilaroi Highway, Narrabri, NSW 2390, Australia

Supplementary data

Figure S1: Descriptive data on the transcriptome of Helicoverpa punctigera: a) GO term classification of the assembled transcripts, b) COG function classification of the assembled transcripts, c) homology statistics of assembled transcripts relative to Blast homology matches at NCBI.

a


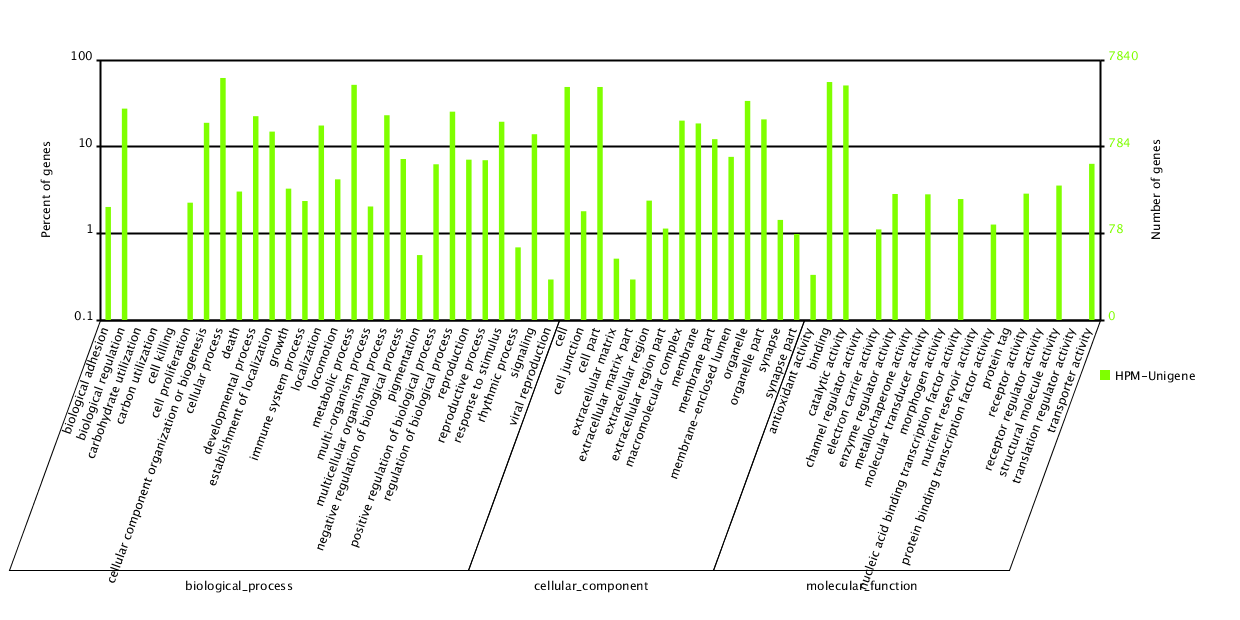


b


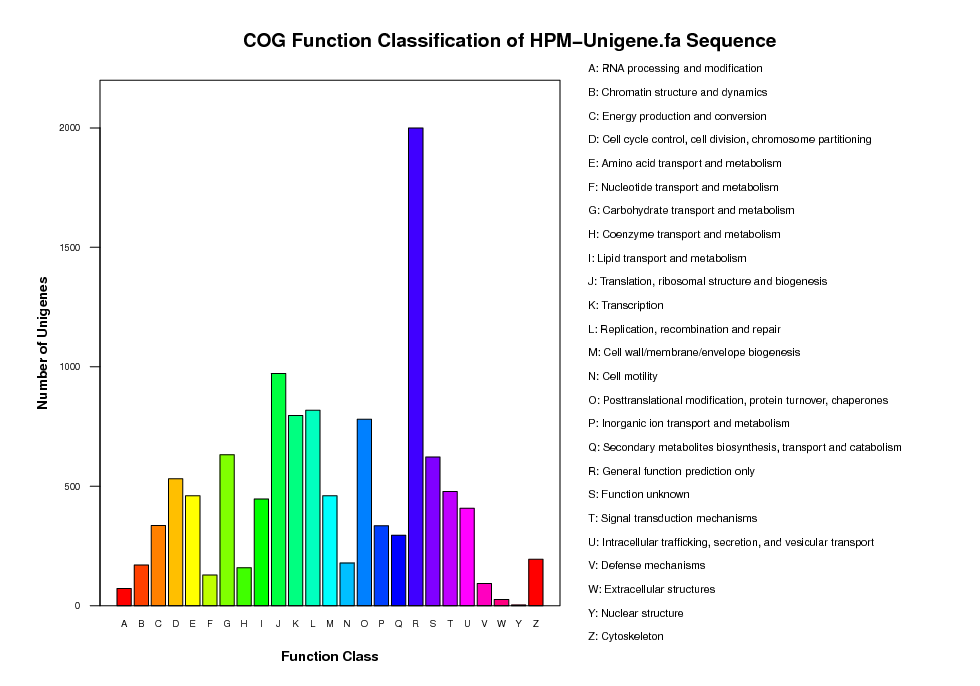


c


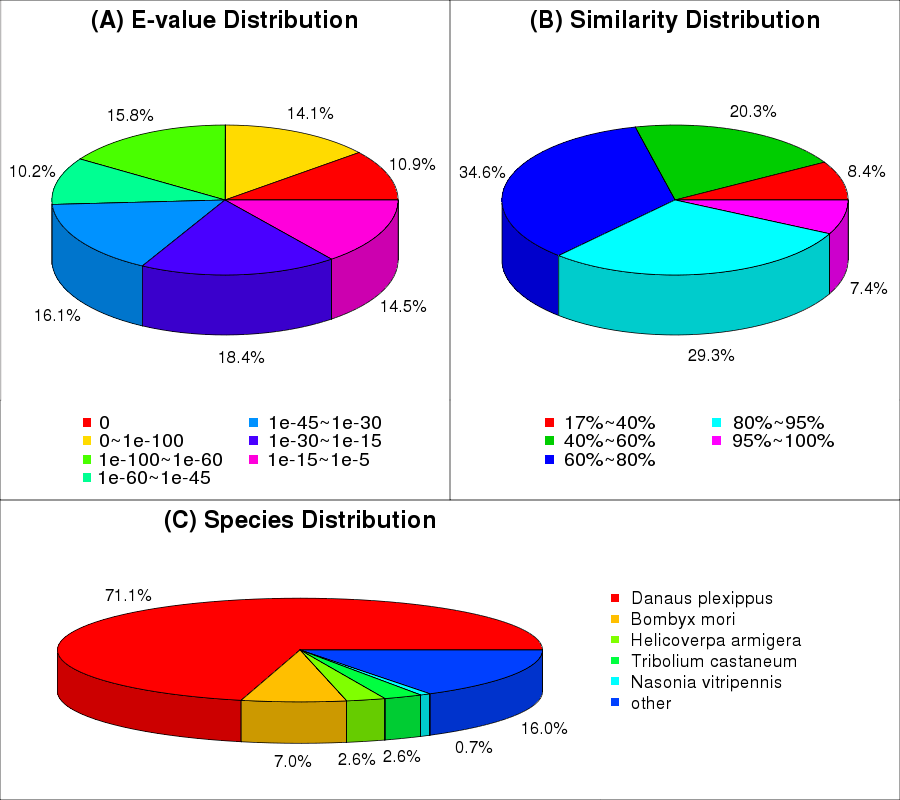


Supplementary Figure 2: Phylogenetic tree of the *Helicoverpa punctigera* cadherin gene compared to other lepidopteran species.


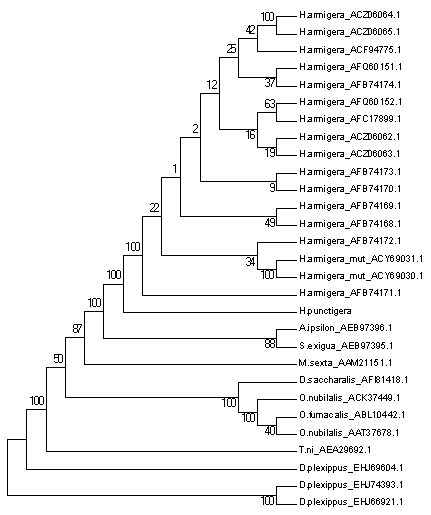


Supplementary figure 3: Amino acid alignment. Highlighted is the binding motif identified in *Manduca sexta* and *Ostrinia nubalis*.

ACZ06064.1_H.armigera MAVDVRIFTAAVFILAAHFTFAQ-DCSYMVAIPRPERPDFPSQNFDGIPWSQYPLIPVEG 59

H.punctigera HPM MAVDVRILTAAVLILAASLSFAQDDCSYMVAIPRPERPDFPSQNFEGVPWSQYPLIPVEE 60

Hp9-3784 MAVDVRILTAAVLILAASLSFAQDDCSYMVAIPRPERPDFPSQNFEGVPWSQNPLIPVED 60

*******:****:**** ::*** *********************:*:**** ******

ACZ06064.1_H.armigera REDVCMNEFEPGNQNPVTVIFMEEEIEGDVAIARLNYRGTNTPIIVSPFSFGTFNMLGPV 119

H.punctigera HPM REDVCMNEFQPDALNPVTVIFMEEEIEGEVAIARLNYRGTNTPTIVTPFSFGTFNMLGPV 120

Hp9-3784 REDVCMNEFQPDALNPVTVIFMEEEIEGDVAIARLNYRGTNTPTIVTPFSFGTFNMLGPV 120

*********:*. **************:************** **:*************

ACZ06064.1_H.armigera IRRIPENGGDWHLVITQRQDYETPGMQQYIFDVRVDDEPLVATVMLLIVNIDDNDPIIQM 179

H.punctigera HPM IRRIPEQGGDWHLVITQRQDFETPGMQQYIFDIRVDDEPLVATVMLLIVNIDDNDPIIQM 180

Hp9-3784 IRRIPEQGGDWHLVITQRQDFETPGMQQYIFDIRVDDEPLVATVMLLIVNIDDNDPIIQM 180

******:*************:***********:***************************

ACZ06064.1_H.armigera FEPCDIPERGETGITSCKYTVSDADGEISTRFMRFEISSDRDDDEYFELVRENIQGQWMY 239

H.punctigera HPM FEPCDIPERGETGITSCKYVVSDADGEISTRFMRFEIESDRNDDDYFELVRENIQGEWMF 240

Hp9-3784 FEPCDIPERGETGITSCKYVVSDADGEISTRFMRFEIESDRNDDEYFELVRENIQGEWMF 240

*******************.*****************.***:**:***********:**:

ACZ06064.1_H.armigera VHMRVHVKKPLDYEENPLHLFRVTAYDSLPNTHTVTMMVQVENVENRPPRWVEIFAVQQF 299

H.punctigera HPM VHMRVHVKKPLDYEENPLHLFRVTAYDSLPNTHTVTMMVQVENVENRPPRWMEIFAVQQF 300

Hp9-3784 VHMRVHVKKPLDYEENPLHLFRVTAYDSLPNTHTVTMMVQVENVENRPPRWMEIFAVQQF 300

***************************************************:********

ACZ06064.1_H.armigera DEKTERSFRVRAIDGDTGIDKPIFYRIETEKGEEDLFSIQTIEGGREGAWFNVAPIDRDT 359

H.punctigera HPM DEKTAQSFRVRAIDGDTGIDKPIFYRIETEEGEEDLFSIQAIEGGREGAWFNVAPIDRDT 360

Hp9-3784 DEKTAQSFRVRAIDGDTGIDKPIFYRIETEEGEEDLFSIQAIEGGREGAWFNVAPIDRDT 360

**** :************************:*********:*******************

ACZ06064.1_H.armigera LEKEVFHVSIIAYKYGDNDVEGSSSFQSKADVVIIVNDVNDQAPLPFREEYSIEIMEETA 419

H.punctigera HPM LEKEVFHVSIIAYKYGDNDVEGSSSFQSKTDVVIIVNDVNDQAPLPFREEYFIEIMEETA 420

Hp9-3784 LEKEVFHVSIIAYKYGDNDVEGSSSFQSKTDVVIIVNDVNDQAPLPFREEYSIEIMEETA 420

*****************************:********************* ********

ACZ06064.1_H.armigera MTLNLEDFGFHDRDLGPHAQYTVHLESIHPPRAHEAFYIAPEVGYQRQSFIMGTQNHHML 479

H.punctigera HPM MTLNLEDFGFHDRDLGPHAQYTVHLESIHPPRAHEAFYIAPEVGYQRQSFIMGTQNHHML 480

Hp9-3784 MTLNLEDFGFHDRDLGPHAQYTVHLESIHPPRAHEAFYIAPEVGYQRQSFI--TQNHHML 478

*************************************************** *******

ACZ06064.1_H.armigera DFEVPEFQNIQLRAIAIDMDDPKWVGIAIINIKLINWNDELPMFESDVQTVSFDETEGAG 539

H.punctigera HPM DFEVPEFQNIQLRAIAIDMDNPKWVGIAIINIKLINWNDELPMFESDVQTVSFDETEGAG 540

Hp9-3784 DFEVPEFQNIQLRAIAIDMDNPKWVGIAIINIKLINWNDELPMFESDVQTVSFDETEGAG 538

********************:***************************************

ACZ06064.1_H.armigera FYVATVVAKDRDVGDKVEHSLMGNAVSYLRIDKETGKIFVTENEAFNYHRQNELFVQIRA 599

H.punctigera HPM FYVATVVAKDRDVDDKVEHSLMGNAVNYLRIDKDTGEIFVTEDEAFNYHRQNELFIQIRA 600

Hp9-3784 FYVATVVAKDRDVDDKVEHSLMGNAVNYLRIDKDTGEIFVTEDEAFNYHRQNELFVQIRA 598

*************.************.******:**:*****:************:****

ACZ06064.1_H.armigera DDTLGEPYNTNTTQLVIKLRDINNTPPTLRLPRSTPSVEENVPDGFVIPTQLNATDPDTT 659

H.punctigera HPM DDTLGEPYNTNTTQLVIQLRDINNTPPTLRLPRVTPSVEENVPDGFVIPTQLDASDPDTT 660

Hp9-3784 DDTLGEPYNTNTTQLVIQLRDINNTPPTLRLPRITPSVEENVPDGFVIPTQLDATDPDTT 658

*****************:*************** ******************:*:*****

ACZ06064.1_H.armigera AELRFEIDWENSYATKQGRNTDSKEYIGCIEIETIYPNINQRGNAIGRVVVREIRDGVTI 719

H.punctigera HPM AELRFEIDWETSYATKQGRNTDPKEFINCIEIVTIYPNINDKGNAIGRVVVREIREHVTI 720

Hp9-3784 AELRFEIDWETSYATKQGRNTDPKEFINCIEIVTIYPNINDKGNAIGRVVVREIREHVTI 718

**********.*********** **:*.**** *******::*************: ***

ACZ06064.1_H.armigera DYEMFEVLYLTVIVRDLNTVIGEDHDISTFTITIIDMNDNPPLWVEGTLTQEFRVREVAA 779

H.punctigera HPM DYEMFEVLYLTVRVRDLNTVIGDDYDVSTFTITIIDMNDNPPLWVEGTLTQEFRVREVAA 780

Hp9-3784 DYEMFEVLYLTVRVRDLNTVIGDDYDISTFTITIIDMNDNPPLWVEGTLTQEFRVREVAA 778

************ *********:*:*:*********************************

ACZ06064.1_H.armigera SGVVIGSVLATDIDGPLYNQVRYTITPRLDTPEDLVDIDFNTGQISVKLHQAIDADEPPR 839

H.punctigera HPM SGVVIGSVLATDIDGPLYNQVRYTITPRLDTPEDLVEIDFNTGQISVKLHQAIDADEPPR 840

Hp9-3784 SGVVIGSVLATDIDGPLYNQVRYTITPRLDTPEDLVEIDFNTGQISVKLHQAIDADEPPR 838

************************************:***********************

ACZ06064.1_H.armigera QNLYYTVIASDKCDLLTVTECPPDPTYFETPGEITIHITDTNNKVPQVEDDKFEATVYIY 899

H.punctigera HPM QHLYYTVVASDKCDLLTVTECPPDPNYFDTPGEITIHITDTNNKVPQVEEDKFDATVYIY 900

Hp9-3784 QHLYYTVIASDKCDLLTVTECPPDPNYFDTPGEITIHITDTNNKVPQVQEDKFDATVYIY 898

*:*****:*****************.**:*******************::***:******

ACZ06064.1_H.armigera EGADDGEHVVQIYASDLDRDEIYHKVSYQINYAINSRLRDFFEMDLETGLVYVNNTAGEL 959

H.punctigera HPM EGADDGERVVQIYASDLDRDEIYHKVSYQINYAINSRLRDFFEIDLETGLVYVNNTAGEL 960

Hp9-3784 EGADDGERVVQIYASDLDRDEIYHKVSYQINYAINSRLRDFFEIDLETGLVYVNNTAGEL 958

*******:***********************************:****************

ACZ06064.1_H.armigera LDRDGDEPTHRIFFNVIDNFYGEGDGNRNQNETQVLVVLLDINDNYPELPETIPWAISES 1019

H.punctigera HPM LDRDGDEPTHRIFFNVIDNFYGEGDGNRNQDETQVLVVLLDINDNYPELPETIPWSISES 1020

Hp9-3784 LDRDGDEPTHRIFFNVIDNFYGEGDGNRNQDETQVLVVLLDINDNYPELPETIPWSISES 1018

******************************:************************:****

ACZ06064.1_H.armigera LEQGERVQPEIFARDRDEPGTDNSRVAYAITGLASTDRDIQMPDLFNMITIERDRGIDQT 1079

H.punctigera HPM LVQGERVEPEIFAPDRDEPGTDNSRVAYAVTGLTITDRDITVPDLFNMITIEKDRGIDQT 1080

Hp9-3784 LVQGERVEPEIFAPDRDEPGTDNSRVAYAVTGLALTDRDITAPHLFNMITIEKDRGIDQT 1078

* *****:***** ***************:***: ***** *.********:*******

ACZ06064.1_H.armigera GILEAAMDLRGYWGTYEIDIQAYDHGIPQRISNQKYPLVIRPYNFHDPVFVFPQPGSTIR 1139

H.punctigera HPM GQLEAAMDLKGYWGTYEIDIQAYDHGVPQRISNQKYPLVIRPYNFHDPVFVFPQPGSTIR 1140

Hp9-3784 GQLEAAMDLRGYWGTYEIDIQAYDHGVPQRISNQKYPLVIRPYNFHDPVFVFPQPGSTIR 1138

* *******:****************:*********************************

ACZ06064.1_H.armigera LAKERAVVNGILATVDGEFLDRIVATDEDGLEAGLVTFSIAGDDEGSQFFDVLNDGVNSG 1199

H.punctigera HPM LAKERAVVNGILATVDGEFLDRIVATDEDGLDAGLVTFSIAGNDEASRFFDVLNDGVNSG 1200

Hp9-3784 LAKERAVVNGILATVDGEFLDRIVATDEDGLEAGLVTFSIAGNDEASRYFDVLNDGVNSG 1198

*******************************:**********:**.*::***********

ACZ06064.1_H.armigera SLTLTRLFPEDFREFQVTIRATDGGTEPGPRSTDCLVTVVFVPTQGEPVFEIRTYTVAFV 1259

H.punctigera HPM ALTITQLFPEDFREFQVTIRATDAGTEPGPRSTDCAVTVVFVPTQGEPVFETSTYTAAFI 1260

Hp9-3784 ALTITQLFPEDFREFQDSGLI------PMKACHTICTT---------------------- 1230

:**:*:********** : * . .*

ACZ06064.1_H.armigera EKDEGMEERAELPRASDPRNIMCEDDCHDTYYSIVGGN-PGEHFRVDPRTNVLTLVRPLD 1318

H.punctigera HPM EQDAGMEERVQLPLAKDPRNIMCEDDCHITYYSIVGGNSPGQHFEVDPLTNVLSLVTPLD 1320

Hp9-3784 -------------------YTYCDDSCYGRWN---------------------------- 1243

*:*.*: :

ACZ06064.1_H.armigera RSEQETHTLIIGASDTPNPAAVLQASTLTVTVNVREANPRPVFQRALYTAGISAGDFIER 1378

H.punctigera HPM RSEQETHTLIIGASDTPNPAAVLQASTLTVTVNVREANPRPVFQRALYTAGISTGDYINR 1380

Hp9-3784 ------------------------------------------------------------ 1243

ACZ06064.1_H.armigera NLLTVVATHSEGLPITYTLIQETMEADPTLEAVQESAFILNPETGVLSLNFQPTAAMHGM 1438

H.punctigera HPM NLLTLLATHSEGLPVTYTLIQESMVADPSLQAVQETAFILNPETGVLSLNFQPTASMHGM 1440

Hp9-3784 ------------------------------------------------------------ 1243

ACZ06064.1_H.armigera FEFEVEATDSRRETARTEVKVYLISDRNRVFFTFNNPLPEVTPQEDFIAETFTAFFGMTC 1498

H.punctigera HPM FEFDVEASDSEGETARTEVKVYLISDRNRVFFTFNNPLNEVTPHEDFIAETFTLFFGMTC 1500

Hp9-3784 ------------------------------------------------------------ 1243

ACZ06064.1_H.armigera NIDRTWWASDPVTGATRDDQTEVRAHLIRDDLPVPAEEIEQLRGNPTLVNSIQRALEEQN 1558

H.punctigera HPM NIDQTLPASDPATGAARDDHTEVRAHFIRDDLPVPAEEIEQLRGNPTLVATIQLALQEQD 1560

Hp9-3784 ------------------------------------------------------------ 1243

ACZ06064.1_H.armigera LQLADLFTGETPILGGDAQARALYALAAVAAALALIVVVLLIVFFVRTRTLNRRLQALSM 1618

H.punctigera HPM LQLADLFTGETPILGGDAQARALYALAGVAAALALLCVILLIVFFVRTRTLNRRLQALSM 1620

Hp9-3784 ------------------------------------------------------------ 1243

ACZ06064.1_H.armigera TKYSSQDSGLNRVGLAAPGTNKHAVEGSNPIWNETLKAPDFDALSEQSYDSDLIGIEDLP 1678

H.punctigera HPM TKYSSQDSGLNRVGLAAPGTNKHAVEGSNPIWNETLKAPDFDALSEQSYDSDLIGIEDLP 1680

Hp9-3784 ------------------------------------------------------------ 1243

ACZ06064.1_H.armigera QFRNDYFPPEEGSSMRGVVNEHVPESIANHNNNFGFNSTPFSPEFANTQFRR 1730

H.punctigera HPM QFRNDYFPPEEGSSMRGVVNEHMPESIANHNNNFGFNATPFSPEFANTQLRR 1732

Hp9-3784 ---------------------------------------------------- 1243

Supplementary figure 4: gDNA – from assembled gDNA seq.

HPM_H.punctigera_gDNA GATGAGGCGTCTCGATTCTTCGACGTGTTGAACGACGGAGTGAACTCGGGCGCCCTCACC

Hp9-3784_gDNA GATGAGGCGTCTCGTTACTTCGACGTGTTGAACGACGGAGTGAACTCGGGCGCCCTCACC

**************:*:*******************************************

HPM_H.punctigera_gDNA ATCACTCAACTCTTCCCTGAAGACTTCCGAGAGTTCCAGGTCAGTGGTCTCATACCAATG

Hp9-3784_gDNA ATCACGCAGCTCTTCCCTGAAGACTTCCGAGAGTTCCAGGACAGTGGTCTCATACCAATG

***** **.*******************************:*******************

HPM_H.punctigera_gDNA CAAGCTTGTCATACGATATGTACGACCTACACCAATT

Hp9-3784_gDNA AAGGCTTGTCATACGATATGTACGACCTACACCTATT

.*.******************************:***

Supplementary Table 1; Primers used in this work

| Sequence 5’ – 3’ | Primer | Purpose |
| --- | --- | --- |
| CCAAGACCAGAACGACCTGA | Hpcad_1_F | RT-PCR and cDNA sequencing |
| AATGAGGAGCATCACCGTGG | Hpcad_1_R | RT-PCR and cDNA sequencing |
| TACGTGATGATCTGCCCGTG | Hpcad_10_F | RT-PCR and cDNA sequencing |
| AAAGTCTGGTGCCTTCAGGG | Hpcad_10_R | RT-PCR and cDNA sequencing |
| CGTCAATGAACACATGCCTGA | Hpcad_11_F | RT-PCR and cDNA sequencing |
| TGCTGACGGCGAAATCAGTA | Hpcad_2_F | RT-PCR and cDNA sequencing |
| TCCTCTCCCTCTTCAGTTTCG | Hpcad_2_R | RT-PCR and cDNA sequencing |
| TCCACGTGTCCATAATAGCGT | Hpcad_3_F | RT-PCR and cDNA sequencing |
| TGGCCCTTAGTTGTATATTCTGAA | Hpcad_3_R | RT-PCR and cDNA sequencing |
| GGCTTCTATGTCGCCACTGT | Hpcad_4_F | RT-PCR and cDNA sequencing |
| TCGATCTCGAAGCGCAGTTC | Hpcad_4_R | RT-PCR and cDNA sequencing |
| CATAAACGACAAAGGCAACGC | Hpcad_5_F | RT-PCR and cDNA sequencing |
| TTGACCCGTGTTGAAGTCGA | Hpcad_5_R | RT-PCR and cDNA sequencing |
| TGTCCACCTGACCCCAACTA | Hpcad_6_F | RT-PCR and cDNA sequencing |
| TCGTCCTGATTACGGTTGCC | Hpcad_6_R | RT-PCR and cDNA sequencing |
| AGAGCCAGAAATCTTCGCCC | Hpcad_7_F | RT-PCR and cDNA sequencing |
| GTCGGTGGCGACTATTCTGT | Hpcad_7_R | RT-PCR and cDNA sequencing |
| TCACCATCACGCAGCTCTTC | Hpcad_8_F | RT-PCR and cDNA sequencing |
| TGTGTGTCTCCTGTTCGCTG | Hpcad_8_R | RT-PCR and cDNA sequencing |
| CTCCACCGGCGATTACATCA | Hpcad_9_F | RT-PCR and cDNA sequencing |
| CGTCATGCCGAAGAACAACG | Hpcad_9_R | RT-PCR and cDNA sequencing |
| CCGACGAGGATGGCTTAGAC | Hp_Cad_diag-F1 |  |
| CGAGTTCACTCCGTCGTTCA | Hp_Cad_diag-R1 |  |
| GGCTTAGACGCTGGACTTGT | Hp_Cad_diag-F2 |  |
| ACTGACCTGGAACTCTCGGA | Hp_Cad_diag-R2 |  |
| TGGCTTAGACGCTGGACTTG | Hp_Cad_diag-F3 |  |
| CGTAGCACGAATCGTCACCT | Hp_Cad_diag-R3 |  |
